# LINC00511 accelerated the process of gastric cancer by targeting miR-625-5p/NFIX axis

**DOI:** 10.1186/s12935-019-1070-0

**Published:** 2019-12-26

**Authors:** Zhaosheng Chen, Honglei Wu, Zhen Zhang, Guangchun Li, Bin Liu

**Affiliations:** Department of Gastroenterology, The Second Hospital of Shandong University, No. 247, Beiyuan Street, Ji Nan, 250033 Shandong China

**Keywords:** LINC00511, miR-625-5p, NFIX, Gastric cancer

## Abstract

**Background:**

Gastric cancer (GC) is a common-sighted cancer which is hard to cure over the world. Substantial researches revealed that long non-coding RNAs (lncRNAs) were fundamental regulators in the process of cancers. Nevertheless, the biological function of LINC00511 and how LINC00511 was involved in the regulatory system in GC remained unclear.

**Methods:**

RIP assays and luciferase reporter assays were performed to illustrate combination between LINC00511 and miR-625-5p. Loss-of-function assays were applied for identifying LINC00511 function in GC.

**Results:**

In our study, LINC00511 was discovered significantly high in expression in GC tissues and cell lines. Moreover, LINC00511 showed a strong expression in I/II and III/IV stage. Knockdown of LINC00511 could inhibit the cell proliferation while enhanced cell apoptosis rate in GC. We used nuclear–cytoplasmic fractionation to judge the subcellular localization of LINC00511. Furthermore, miR-625-5p was found to have binding sites for LINC00511 and negatively regulated by LINC00511. Overexpression of miR-625-5p repressed the course of GC. And knockdown of miR-625-5p could recover the effects of LINC00511 silence. Besides, NFIX was discovered as a downstream target of miR-625-5p and overexpression of NFIX could offset the influence of LINC00511 silence. The results of vivo studies manifested that down-regulation of LINC00511 could reduce the Ki67 expression and NFIX while lifted the expression of miR-625-5p.

**Conclusion:**

Overall, the results from our study demonstrated that LINC00511 could function as a tumor promoter by targeting miR-625-5p NFIX axis, suggesting LINC00511 could be considered as a target for GC treatment.

## Background

Gastric cancer (GC) is a cancer which brought pains to patients mind and body with high occurrence rates [1], while patients in early stage of GC might be cured thoroughly with the efficient methods [2]. So far, though the chemotherapy and radiotherapy were applied in the treatment, the recurrence rate was still extremely poor due to metastasis and the process of these treatments brought much pain to patient physically and psychologically [3]. It is urgently needed to discover a novel treatment target to solve this problem.

Mounting articles indicated that long non-coding RNAs (lncRNAs) were actively participating in the process of cancers [4]. And the abnormal expression of lncRNAs could function as tumor inhibitors or promoters to regulate the biological activities in cancers [5, 6]. It was found that overexpression of PANDAR predicted poor prognosis and facilitated cell proliferation in cervical cancer [7]. CCAT1 was discovered to elevate radiosensitivity by sponging miR-148b in breast cancer [8]. CASC9 overexpression was seen as a tumor promoter in esophageal squamous cell carcinoma by negatively mediating PDCD4 via EZH2 [9]. However, the role of LINC00511 in GC was unknown to us.

So far, accumulating researches introduced competing endogenous RNA (ceRNA) regulatory system where lncRNAs were treated as rivals to mRNAs to bind with microRNAs (miRNAs) so that those mRNAs lost their ability to translate into protein [10–12]. H19 was reported as a ceRNA to bind to miR-29b-3p in bladder cancer to affect epithelial mesenchymal transition (EMT) [13]. UCA1 was seen as a ceRNA of Sox4 in esophageal cancer to promote cell proliferation [14]. HNF1A-AS1 was regarded as a ceRNA to accelerate the metastasis of colon cancer repressing miR-34a/SIRT1/p53 feedback loop [15]. MiRNAs were a group of non-coding RNAs which were about 18–25 nucleotides in length. Recently, miRNAs have been reported to exert their function in multiple cancers [16, 17]. miR-195 was seen as a tumor suppressor in non-small cell lung cancer through targeting CHEK1 [18]. Nevertheless, how miR-625-5p took part in the course of GC needed to be explained. In our study, LINC00511 functioned as a ceRNA of miR-625-5p and fostered the progression of GC by targeting miR-625-5p/NFIX axis.

Our study was designed to explore the function of LINC00511 in GC cells. The results of our study manifested that knockdown of LINC00511 could successfully hinder the progression of GC by targeting miR-625-5p/NFIX axis, hinting that LINC00511 could be taken into account for GC treatment.

## Materials and methods

### Human tissue samples

Gastric cancer specimens and adjacent normal tissues of 35 participants were gathered from June 2013 to August 2018 from the Second Hospital of Shandong University. Before surgery, no treatment was applied in each patient. The study was allowed by the Institutional Review Committee and every patient signed informed consent before resection. When the surgical resection was finished, tissues were frozen instantly in liquid nitrogen, then removed and long-stored in a refrigerator at − 80 °C.

### Cell culture

Human gastric mucosa epithelial cell (GES1) and gastric cancer cells (HGC27, BGC823, MGC803 and SGC7901) were bought from Chinese Academy of Sciences (Beijing, China). The above cells were incubated in DMEM (Gibco-BRL, Grand Island, NY, USA) containing 10% FBS (Gibco-BRL) plus 1% penicillin and streptomycin (Invitrogen, Carlsbad, CA, USA). Cells were developed in a humidified condition with 5% CO_2_ at 37 °C. Medium for incubation was changed every 3 days.

### Cell transfection

Transfection of HGC27 or BGC823 cells was processed in line with the protocol of Lipofectamine 3000 kit (Invitrogen). The duplex specific short hairpin RNAs (shRNAs) to LINC00511 (termed sh-LINC00511#1 and sh-LINC00511#2) and their corresponding nonspecific shRNAs as negative control (NC; termed sh-NC), were designed and synthesized by Genechem (Shanghai, China). The targeting sequences of LINC00511-specific shRNAs were as follows: sh-LINC00511#1, 5′-TACCGCGACACAAGTCTCCGTCCTCCTT-3′; sh-LINC00511#2, 5′-TCCGATGACGGGAGACGGGGTTCTGTCC-3′; sh-NC, 5′-TACCTCTGGATTCTCTCCCGCTCTCTGA-3′. For overexpressing LINC00511 or NFIX, cells were transfected with the pcDNA3.1 vector targeting LINC00511 or NFIX for 48 h, with cells treated with the empty pcDNA3.1 vector (Genechem) used as NC. The overexpression and silencing of miR-625-5p was achieved by transfecting cells with miR-625-5p mimics/inhibitors or NC mimics/inhibitors (all, Gene Pharmacy, Shanghai, China). 48 h post-transfection, cells were reaped for subsequent analysis.

### Quantitative real-time PCR

Total RNA was obtained by TRIzol reagent (Invitrogen). Total RNA was reverse-transcribed into cDNA by use of a Reverse Transcription Kit (TaKaRa, Dalian, China). Then, qRT-PCR was progressed in Bio-Rad CFX96 (Bio-Rad, Hercules, CA, USA) by using a SYBR-Green Real-Time PCR Kit (Takara). 2^− ∆∆Ct^ method was chosen for calculating fold expression changes. In addition, GAPDH/U6 was the internal reference.

### CCK-8 assay

Transfected HGC27 or BGC823 cells were inoculated in fresh 96-well plates and cultured over specific time points (0, 24, 48, 72 and 96 h), followed by incubation with CCK-8 solution (Dojindo, Kumamoto, Japan) for another 4 h. Finally, the absorbance was measured under a microplate reader (Bio-Tek Instruments, Hopkinton, MA, USA) at 450 nm. Bio-triple repeats were required for CCK-8 assay.

### TUNEL assay

Cell apoptosis of transfected HGC27 or BGC823 cells was determined by TUNEL apoptosis kit (Roche, Mannheim, Germany). Biologic coloring agent of DAPI (Haoran, Shanghai, China) was applied for dying the above cells. An EVOS FL microscope (Invitrogen) was employed for detecting the relative fluorescence intensity. Bio-triple repeats were required for TUNEL assay.

### Colony formation assay

Transfected HGC27 or BGC823 cells were cultured in each well of a 6-well plate. After 2 weeks of cultivation, colonies were fixed in 4% PFA (Solarbio, Beijing, China) for 10 min and dyed with crystal violet (Solarbio) for 5 min. Colonies were counted manually later. Bio-triple repeats were required for colony formation assay.

### Flow cytometer

The Annexin V/PI staining apoptosis kit (BD Biosciences, Franklin Lakes, NJ, USA) was applied for measuring the proportion of apoptotic cells. Transfected HGC27 or BGC823 cells were loaded in 6-well plates, after which were washed twice using PBS (Sigma-Aldrich, St. Louis, MO, USA) and re-suspended in 1X Annexin V binding buffer (Invitrogen). Thereafter, cells were added to Annexin V-FITC and PI in a tube to incubate for 15 min. 400 µL of 1 ×  binding buffer was subsequently supplemented to each tube, followed by implementation of flow cytometry within 1 h. Final fluorescence was obtained on BD FACSCANTO II flow cytometer (BD Biosciences). Bio-triple repeats were required for flow cytometry analysis.

### Western blot

Western blot was carried out in the light of prior method [19]. Primary antibodies against cleaved caspase-3 (ab2302), total caspase-3 (ab13847), Bax (ab32503), Bcl-2 (ab32124), NFIX (ab101341) and GAPDH (ab9485) were all gained from Abcam (Cambridge, USA) and applied individually. Bio-triple repeats were required for western blotting.

### Subcellular fractionation

The cytoplasmic and nuclear RNA purification kit (Norgen, Ontario, Canada) was employed for separating and purifying cytoplasmic and nuclear RNA based on specifications. Expression levels of LINC00511, GAPDH (cytoplasmic control) and U6 (nuclear control) were examined using qRT-PCR assays.

### RNA immunoprecipitation (RIP)

RIP was undertaken employing the Magna RIP RNA-binding protein immunoprecipitation kit (Millipore, Billerica, MA, USA). HGC27 or BGC823 cells were re-suspended in RIP lysis buffer, followed by incubation with RIP buffer containing magnetic beads (Invitrogen) conjugated with anti-Ago2 antibody (Abcam) or anti-IgG antibody (Abcam) and rotated at 4  °C overnight. The abundance of LINC00511 or miR-625-5p or NFIX was assayed by qRT-PCR.

### Luciferase reporter assay

LINC00511-WT/MUT or NFIX-WT/MUT was sub-cloned into the pmirGLO dual-luciferase vector (Promega, Madison, WI, USA) so as to generate pmirGLO-LINC00511-WT/MUT or pmirGLO-NFIX-WT/MUT. The pmirGLO-LINC00511-WT/MUT was co-transfected into HGC27 or BGC823 cells with miR-625-5p mimics or NC mimics. The pmirGLO-NFIX-WT/MUT was co-transfected into HGC27 or BGC823 cells with miR-625-5p mimics or miR-625-5p mimics + pcDNA3.1/LINC00511 or NC mimics. Following co-transfection for 48 h, dual luciferase reporter assay system (Promega) was applied.

### RNA pull-down

Cell lysates of HGC27 or BGC823 cells were incubated with Bio-miR-625-5p-WT/MUT or Bio-NC. Magnetic beads were later added. At length, qRT-PCR was undertaken for assaying.

### Xenograft models

The nude mice were gained from Vital River Laboratory (VRL, Beijing, China). GC cells transfected with sh-LINC00511#1 and sh-NC were reaped. Afterward, cells were injected into mice, subcutaneously. Volume of tumor was recorded by measuring length and width every 4 days. 4 weeks later, mice were killed, and the tumor weight was taken down.

### Immunohistochemistry (IHC)

Tumor tissues obtained from xenograft models were embedded in paraffin (Sigma-Aldrich) and subjected to IHC. Tissues were deparaffinized and hydrated, followed by permeabilized for 10 min using 0.5% Triton X-100 (Sigma-Aldrich) in PBS. IHC for anti-Ki67 (Abcam) was carried out by the indirect avidin biotin-enhanced horseradish peroxidase method (Vector Laboratories, Burlingame, CA, USA). Sections were observed via a microscope (Nikon, Tokyo, Japan) and assayed using the Image-Pro Premier software offline program (Media Cybernetics, Rockville, MD, USA).

### Statistical analysis

Data were denoted as mean ± SD. GraphPad Prism 7 software package (GraphPad Software, La Jolla, CA, USA) was used for analyzing the experimental data. One-way ANOVA or Student’s t-test was used to confirm the difference in two or multiple groups. *P  *< * 0.05* had statistically significance. Most importantly, this experiment was made in triplicate.

## Results

### LINC00511 was found high expressed in GC tissues and cells

**Fig. 1:**
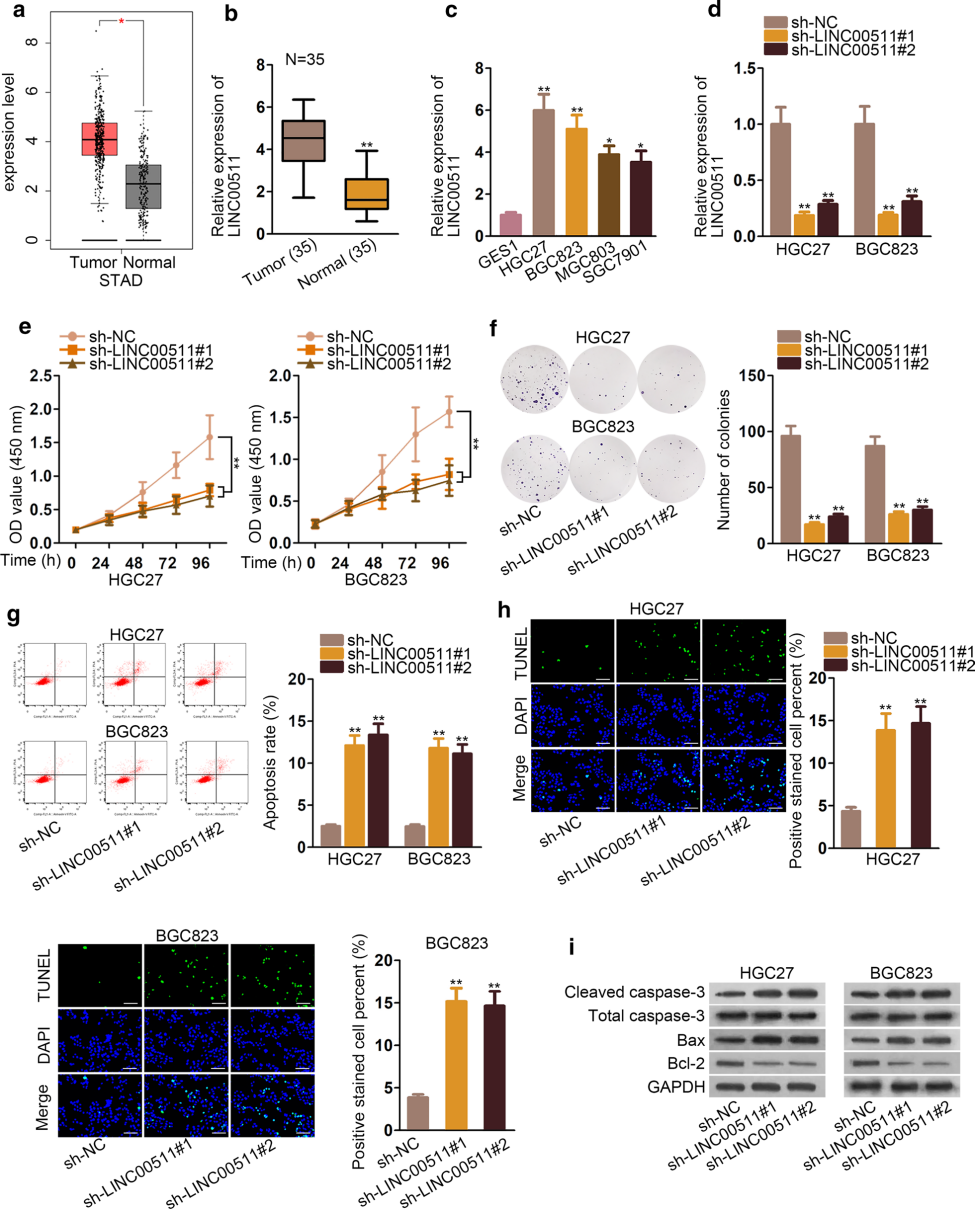
LINC00511 was found high expressed in GC tissues and cells **a** TCGA database showed the expression of LINC00511 in GC tissues and normal adjacent tissue. **b** The expression of LINC00511 in GC tissues and normal adjacent tissue was examined by qRT-PCR assays. **c** LINC00511 expression was tested in GC cell lines (HGC27, BGC823, MGC803 and SGC7901) and normal gastric epithelial cell (GES1) examined by qRT-PCR assay. **d** The expression of LINC00511 was examined by qRT-PCR assay transfected with sh-LINC00511#1, #2. **e**, **f** CCK8 and colony formation assays detected the cell proliferation. **g**, **h** Flow cytometry analysis and TUNEL assays were conducted to appraise apoptosis rate. **i** Western blot assays were performed to measure the relevant protein of apoptosis. *P < 0.05, **P < 0.01

To investigate the function of LINC00511 in GC, first, we searched the TCGA database and found that the expression of LINC00511 was significantly high in GC tissues (Fig. 1a). Then, we detected LINC00511 expression in 35 pairs GC tissues and adjacent normal tissue and the results displayed that LINC00511 was conspicuously high expressed in GC tissues (Fig. 1b). Subsequently, we evaluated the expression of LINC00511 in I/II and III/IV stage. The data of qRT-PCR revealed that LINC00511 expression was overtly strong in I/II and III/IV. But no distinct changes could be seen between them (Additional file 1: Fig. S1a). QRT-PCR assays were applied to test LINC00511 expression in GC cell lines (HGC27, BGC823, MGC803 and SGC7901) and normal gastric epithelial cell (GES1). The results exhibited that LINC00511 expression was remarkably high in GC cell lines (Fig. 1c). Then, we transfected sh-LINC00511#1 and sh-LINC00511#2 into HGC27 and BGC823 cells and used qRT-PCR assays to appraise the expression of LINC00511. The outcome showcased that the expression of LINC00511 was declined dramatically (Fig. 1d). Functional assays were conducted to examine the effects of LINC00511 on GC growth. The effects of LINC00511 knockdown repressed the cell proliferation in CCK8 and colony formation assay (Fig. 1e, f). The rate of apoptosis was enhanced by knockdown of LINC00511 in flow cytometry analysis and TUNEL assay (Fig. 1g, h). The associated proteins of apoptosis were measured by western blot and the consequence revealed that cleaved caspase-3 and Bax expression were increased by knockdown of LINC00511 while Bcl-2 expression was cut down strikingly (Fig. 1i). In short, LINC00511 was high expressed in GC tissues and cell lines and knockdown of LINC00511 suppressed the GC cell proliferation while promoted apoptosis.

### LINC00511 bound to miR-625-5p

**Fig. 2:**
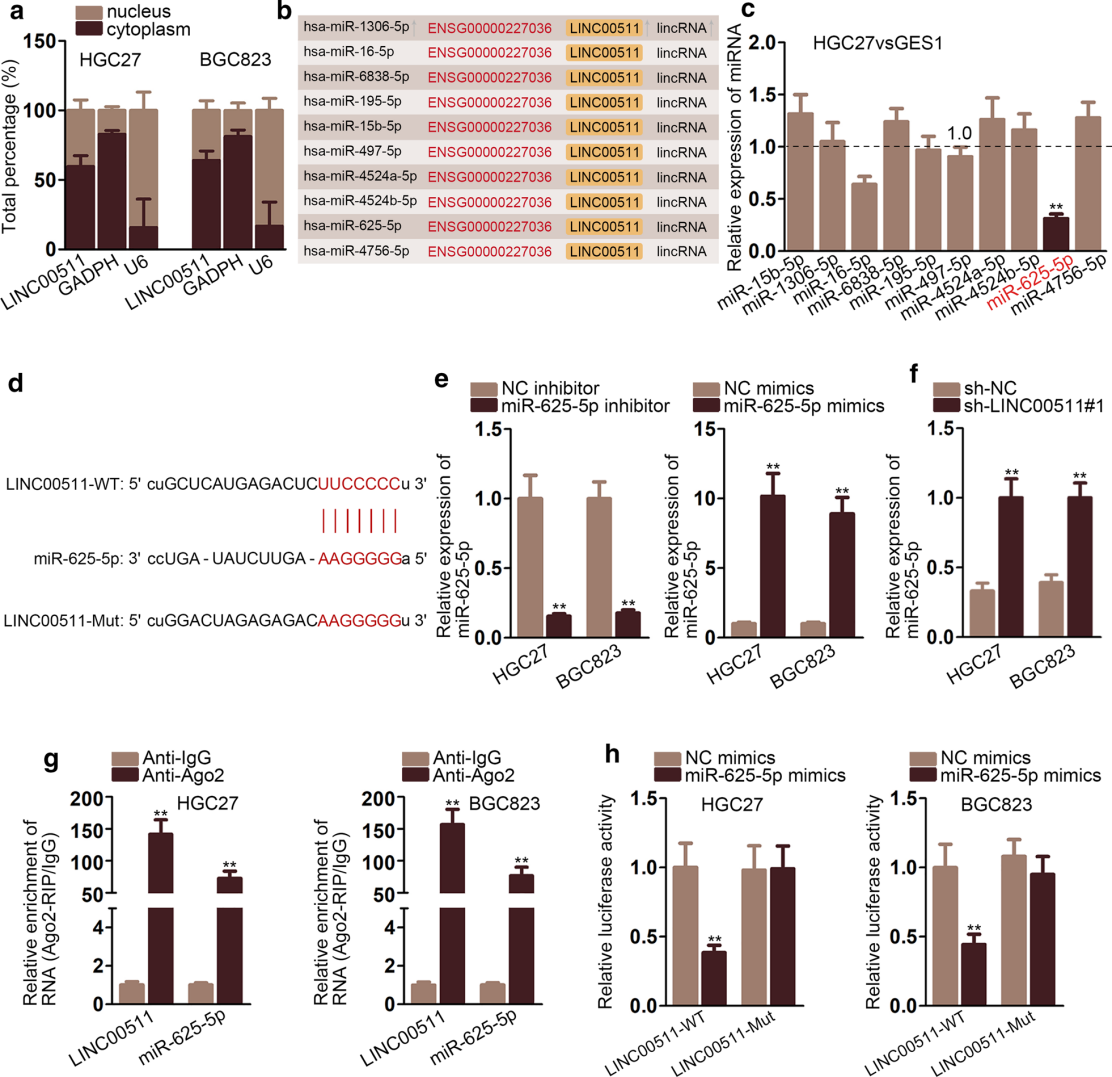
LINC00511 bound to miR-625-5p **a** Nuclear–cytoplasmic fractionation was conducted to examine the distribution of LINC00941 nucleolus and cytoplasm. **b** Data from starBase database predicted possible miRNAs binding with LINC00511. **c** QRT-PCR assay was carried out to test the expression of miRNAs in GC cell lines. **d** The potential binding sites between miR-625-5p and LINC00511 was projected by bioinformatics. **e** The expression of miR-625-5p was tested in HGC27 and BGC823 cells transfected with miR-625-5p inhibitor and miR-625-5p mimics. **f** QRT-PCR assays were used to test the expression of miR-625-5p in cells transfected with sh-LINC00511#1. **g** RIP assays were carried out to illustrate that miR-625-5p and LINC00511 coexisted in the RISCs. **h** Luciferase reporter assays proved LINC00511 could bind to miR-625-5p. *P < 0.05, **P < 0.01

To explore the function of LINC00511 in ceRNA network, nuclear–cytoplasmic fractionation assay was carried out and the results depicted that LINC00511 amassed in cytoplasmic (Fig. 2a). Then, we used starBase database to search miRNAs which had binding sites with LINC00511 (Fig. 2b). QRT-PCR assays were carried out to evaluate the expression of these miRNAs in GC cell lines and the results delineated that miR-625-5p expression was significantly lowered in GC cell lines (Fig. 2c). The putative binding sites between LINC00511 and miR-625-5p were predicted by bioinformatics (Fig. 2d). The expression of miR-625-5p was tested by qRT-PCR assays in HGC27 and BGC823 cells treated with miR-625-5p inhibitor and miR-625-5p mimics. The outcomes delineated that the expression of miR-625-5p was increased by miR-625-5p mimics while dropped by miR-625-5p inhibitor (Fig. 2e). Subsequently, sh-LINC00511#1 was transfected into cells and qRT-PCR assays were used to appraise the expression of miR-625-5p. The results displayed that miR-625-5p expression was lifted by sh-LINC00511#1 (Fig. 2f). Then, RIP assays were conducted and we could observe that both LINC00511 and miR-625-5p were enriched in Ago2 while no distinct change could be found in IgG. The results confirmed that LINC00511 and miR-625-5p coexisted in RNA-induced silencing complexes (RISCs) (Fig. 2g). Furthermore, luciferase reporter assays were conducted to illustrate whether miR-625-5p could bind to LINC00511. The consequence disclosed that the activity of plasmid built with LINC00511-WT was decreased by miR-625-5p mimics prominently while no notable change could be found in plasmid set with LINC00511-Mut (Fig. 2h). Thus, LINC00511 could bind to miR-625-5p. In short, LINC00511 bound to miR-625-5p and negatively regulated miR-625-5p.

### LINC00511 accelerated the course of GC via declining miR-625-5p expression

**Fig. 3:**
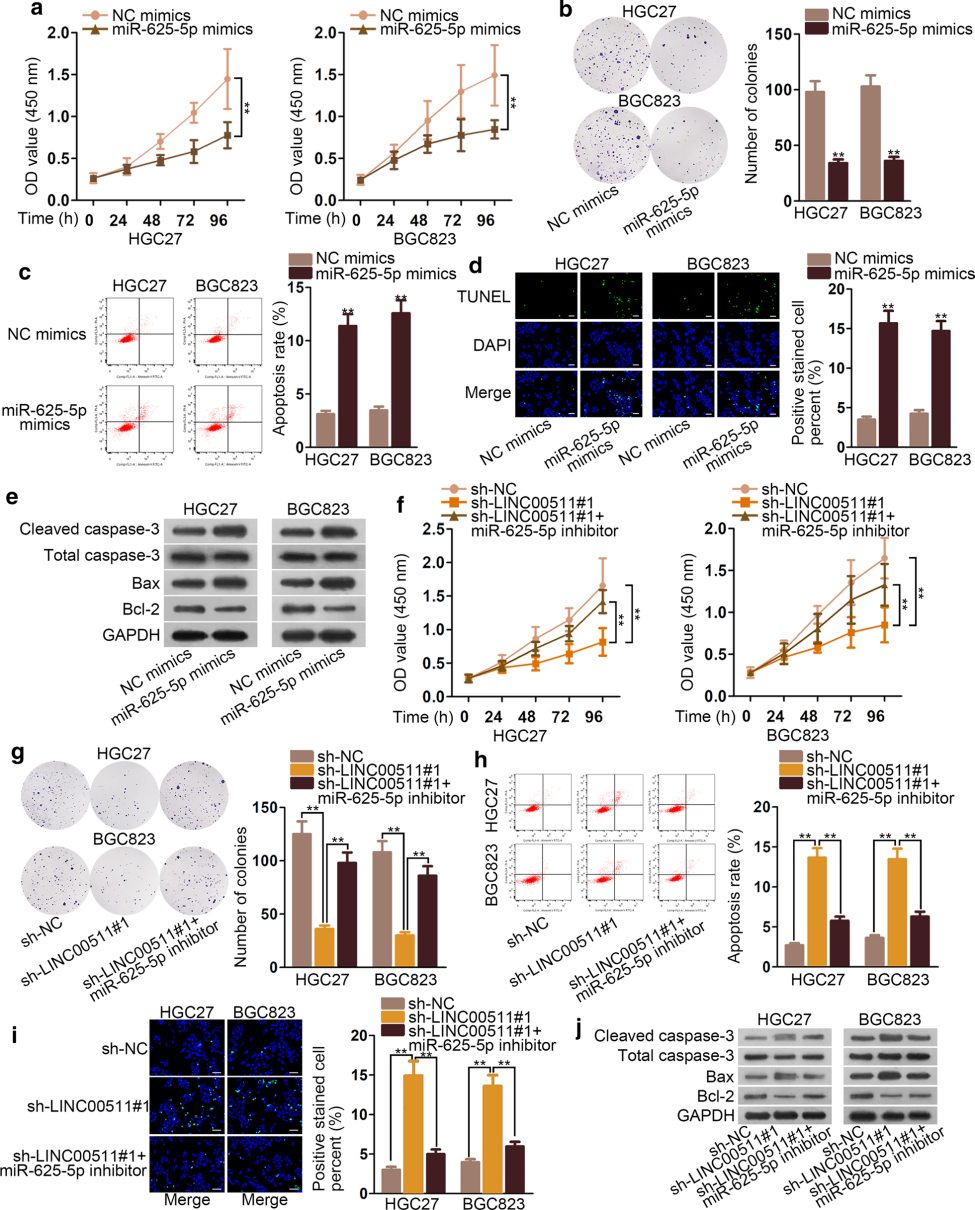
LINC00511 accelerated the course of GC via declining miR-625-5p expression **a**, **b** CCK8 and colony formation assay were performed to estimate the cell proliferation in cells transfected with miR-625-5p mimics. **c**, **d** Flow cytometry analysis and TUNEL assays were conducted to measure apoptosis in cells transfected with miR-625-5p mimics. **e** Western blot assays were used to measure the relevant protein of apoptosis. **f**, **g** The effect of miR-625-5p inhibitor and sh-LINC00511#1 were examined by CCK8 and colony formation assay on cell proliferation. **h**, **i** The influence of miR-625-5p inhibitor and sh-LINC00511#1 were appraised on the rate of apoptosis in flow cytometry analysis and TUNEL assay. **j** Relevant proteins of apoptosis were tested in western blot in cells transfected with miR-625-5p inhibitor and sh-LINC00511#1. *P < 0.05, **P < 0.01

MiR-625-5p mimics were transfected into GC cell lines to verify how miR-625-5p affected the progression of GC. As the results exhibited in CCK8 and colony formation assays, the ability of cell proliferation was lessened by miR-625-5p mimics (Fig. 3a, b). However, the rate of apoptosis was lifted by miR-625-5p overexpression (Fig. 3c, d). The relevant proteins level of apoptosis revealed that Bax and cleaved caspase-3 expression were inflated while Bcl-2 expression was reduced by miR-625-5p mimics (Fig. 3e). Then, we explored how LINC00511 functioned as a sponge of miR-625-5p in GC cells. Both sh-LINC00511#1 and miR-625-5p inhibitor were transfected into HGC27 and BGC823 cells and the results disclosed that miR-625-5p down-regulation could recover the effects of sh-LINC00511#1 on the capacity of proliferation and apoptosis in GC cells (Fig. 3f–j). In a word, miR-625-5p mimics hindered the GC growth and miR-625-5p inhibitor could counteract the results made by knockdown of LINC00511.

### NFIX was the downstream target of miR-625-5p

**Fig. 4:**
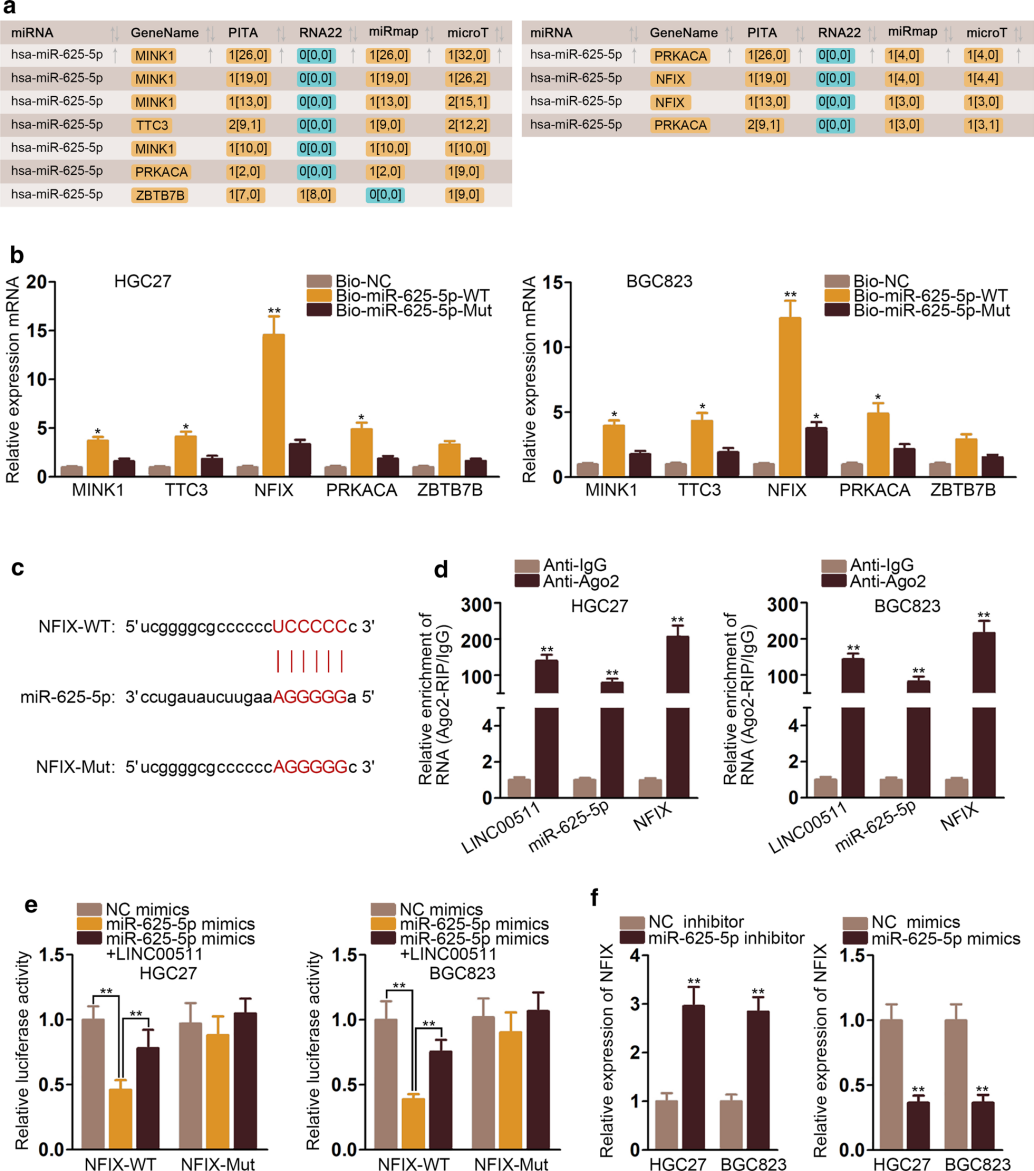
NFIX was the downstream target of miR-625-5p **a** StarBase projected potential mRNAs that could bind to miR-625-5p. **b** RNA pull down assays were conducted that NFIX combines most with miR-625-5p. **c** Bioinformatics projected the putative binding sites between miR-625-5p and NFIX. **d** RIP assays were applied to verify that LINC00511, miR-625-5p and NFIX coexisted in RISCs. **e** Luciferase reporter assays were performed to confirm that LINC00511 competitively bound to miR-625-5p with NFIX. **f** The expression of NFIX was examined in the cells transfected with miR-625-5p inhibitor and miR-625-5p mimics. *P < 0.05, **P < 0.01

To further analyze how LINC00511 regulated the GC growth, we used starBase to discover the downstream target of miR-625-5p and the outcome was displayed in Fig. 4a. Then, RNA pull down assays were carried out and the consequence revealed that NFIX could bind most to miR-625-5p while no evident sign could be seen in other mRNAs (Fig. 4b). Bioinformatics projected the binding sites between miR-625-5p and NFIX (Fig. 4c). RIP assays were conducted and the results verified that LINC00511, miR-625-5p and NFIX all coexisted in RISCs (Fig. 4d). Moreover, luciferase reporter assays were carried out and the outcome displayed that LINC00511 could bind to miR-625-5p competitively against NFIX so that the activity of plasmid was regained (Fig. 4e). MiR-625-5p mimics and miR-625-5p inhibitor were transfected into cells and RT-qPCR assays were used to evaluate the expression of NFIX. The outcome manifested that NFIX expression was enhanced by miR-625-5p inhibitor while decreased by miR-625-5p mimics (Fig. 4f). In brief, NFIX was the downstream target of miR-625-5p and was negatively regulated by miR-625-5p.

### LINC00511 fostered the process of GC via targeting miR-625-5p/NFIX axis

**Fig. 5:**
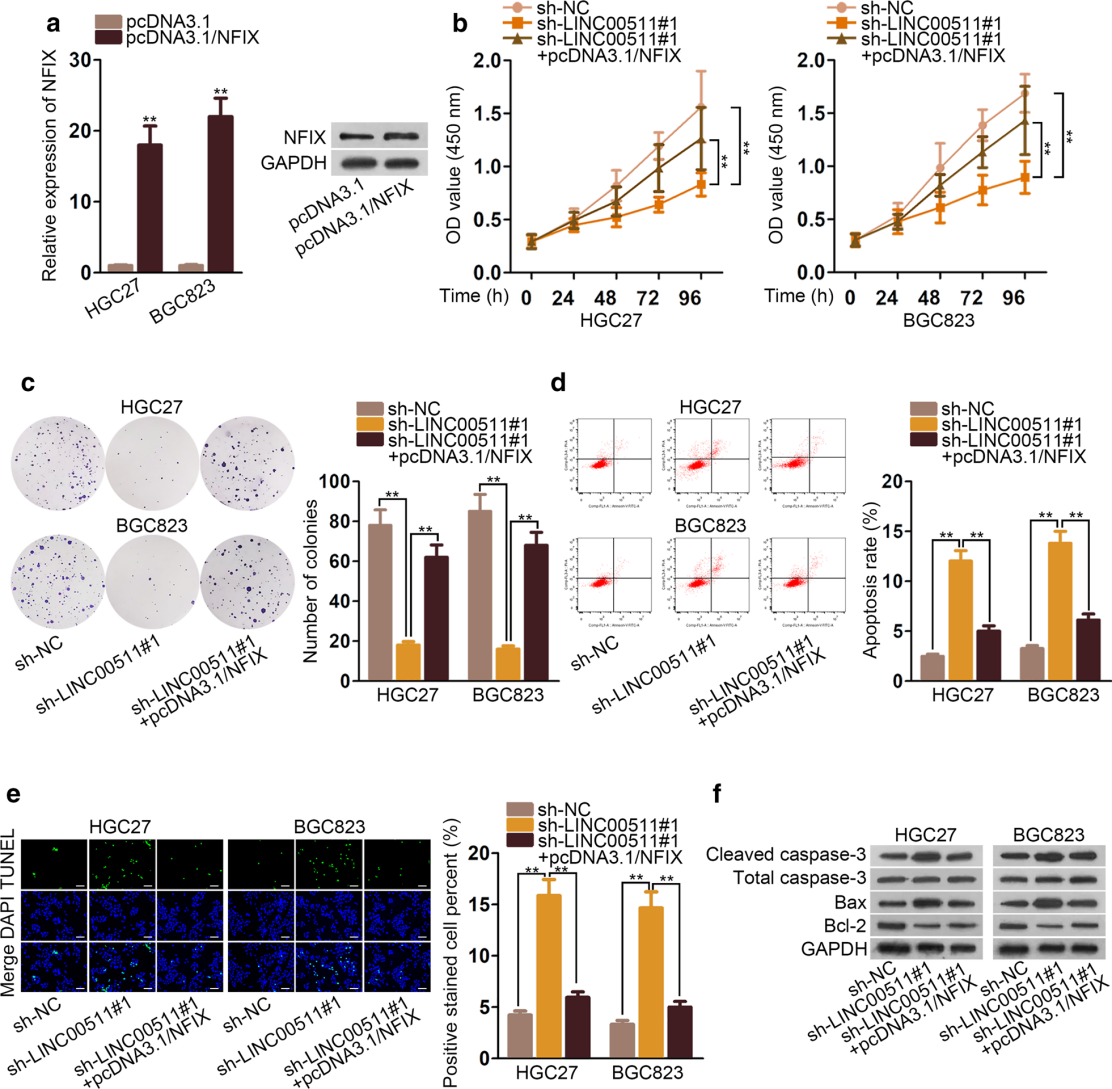
LINC00511 fostered the process of GC via targeting miR-625-5p/NFIX axis **a** RT-qPCR assays and western blot assays were conducted to detect the expression of NFIX in cells transfected with pcDNA3.1/NFIX. **b**, **c** The rescue effect of pcDNA3.1/NFIX on cell proliferation was tested in CCK8 and colony formation assay. **d**, **e** The rescue influence of pcDNA3.1/NFIX was evaluated on rate of apoptosis in flow cytometry analysis and TUNEL assays. **f** The rescue effect of pcDNA3.1/NFIX on protein in apoptosis was appraised in western blot. *P < 0.05, **P < 0.01

To illustrate the effectiveness of LINC00511/miR-625-5p/NFIX axis in GC cells, first, we detected the expression of NFIX in cell transfected with pcDNA3.1/NFIX. And the results examined by qRT-PCR assays and western blot depicted that NFIX expression was lifted by pcDNA3.1/NFIX (Fig. 5a). The effects of sh-LINC00511#1 on capacity of cell viability were offset by pcDNA3.1/NFIX in CCK8 and colony formation assay (Fig. 5b, c). The rate of apoptosis was inflated by knockdown of LINC00511, which could be countervailed by overexpression of NFIX (Fig. 5d, e). The same result could be found in the expression of relevant proteins in apoptosis process (Fig. 5f). In conclusion, the influence caused by knockdown of LINC00511 was regained by overexpression of NFIX.

### LINC00511 silence restricted tumor growth in vivo in GC cells

**Fig. 6:**
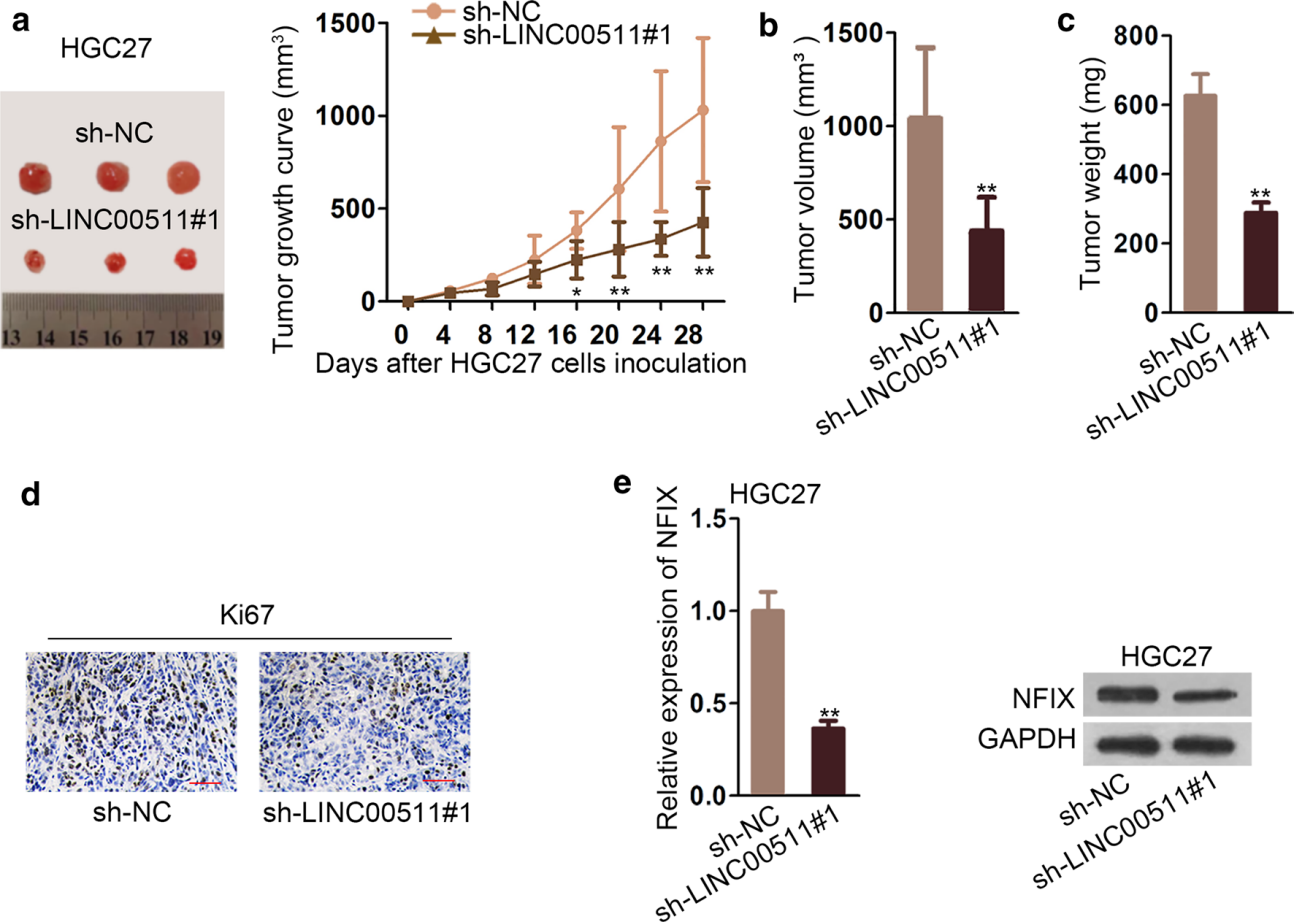
LINC00511 silenced restricted tumor growth in vivo in GC cells **a** Tumor images**. b** The effect of LINC00511 knockdown was examined on tumor growth. **c** The result of tumor volume affected by sh-LINC000511#1. **d** Tumor weight in cells transfected with sh-LINC00511#1 and sh-NC. **e** The expression of Ki67 was tested by immunohistochemistry. **f** QRT-PCR assays and western blot were used to measure NFIX and protein. *P < 0.05, **P < 0.01

To get a deep understanding of how LINC00511 affected the ability of tumor proliferation in GC cells, sh-LINC00511#1 was transfected into HGC27 cells and then transplanted it into nude mice. The tumor pictures were displayed in Fig. 6a. The results displayed that sh-LINC00511#1 obviously diminished tumor growth in comparison with normal control (Fig. 6b–d). The expression of Ki67 was measured by IHC in cells and the results delineated that Ki67 expression was declined by sh-LINC00511#1 (Fig. 6e). Western blot and qRT-PCR assays were performed to test the expression of NFIX and the protein. The consequence revealed that NFIX expression was cut down by sh-LINC00511#1 (Fig. 6f). The data of qRT-PCR revealed that miR-625-5p expression was enhanced by down-regulation of LINC00511 (Additional file 1: Fig. S1b). To sum up, knockdown of LINC00511 constricted the tumor growth of GC.

## Discussion

GC brings a lot of pain and trouble to patients and it is necessary to cure it in early stage. When it developed into end-stage, the recurrence rate of treatment became increased due to metastasis [20, 21]. Thus, early diagnosis is extremely vital for the treatment of GC. Recently, emerging studies surrounded the topic of ceRNA regulatory system where lncRNAs would bind to miRNAs competitively against mRNAs. Then, mRNAs could be translated into proteins and play biological function in in physiological and pathological process [22, 23]. It was observed that ROR played as a ceRNA to modulate Oct4, Sox2 and Nanog expression by sponging miR-145 in colonic cancer stem cell [24]. The GBAP1 pseudogene worked as a ceRNA via sponging miR-22-3p [25]. In a word, these examples verified that lncRNAs modulated the development of cancer through ceRNA system.

In our study, LINC00511 was discovered highly expressed in GC tissues and cells, which had the same results as it was shown in osteosarcoma [26]. Then, we detected the expression of LINC00511 in I/II and III/IV stage. The outcomes revealed that LINIC00511 was powerful in expression among I/II and III/IV stage but there is no striking difference between them. Knockdown of LINC00511 could repress cell proliferation while promoted apoptosis in GC. Based on these data, LINC00511 was verified to have crucial functions in GC progression. But these data were not sufficient to illustrate it did exert tremendous effects in early stage of GC. The relationship between LINC00511 and early development of GC would be explored in our future research. Besides, nuclear–cytoplasmic fractionation was used and the results showcased that LINC00511 amassed in cytoplasmic. A recent research also manifested that LINC00511 localized in cytoplasm in pancreatic adenocarcinoma [27]. And with the help of starBase database, we found that some miRNAs had binding sites with LINC00511. And qRT-PCR assays were used to detect these miRNAs expression and we found that miR-625-5p was in a low level of expression in GC cell lines. The previous study confirmed that miR-625-5p was a tumor inhibitor in glioma [28]. Moreover, overexpression of miR-625-5p could suppress the cell proliferation while enhance apoptosis of GC. LINC00511 was reported to function as a ceRNA by sponging miR-29b-3p in previous study. While we found that LINC00511 served as a sponge of miR-625-5p and miR-625-5p inhibitor could recover the effect of sh-LINC00511#1. Subsequently, we used starBase database to find out the downstream target of miR-625-5p and the results of RNA pull down assays manifested that NFIX bound to miR-625-5p evidently while others depicted no conspicuous change. So, NFIX was selected as the target of miR-625-5p. NFIX was introduced to have oncogenic function [29]. Then, the results of rescue assay proved the effectiveness of LINC00511/miR-625-5p/NFIX axis in the course of GC cell. Overexpression of NFIX could counteract the effects of LINC00511-induced on cell proliferation and apoptosis. These results strongly confirmed LINC00511 role in ceRNA system in GC cell.

To further understand the role of LINC00511 in GC cell, we also conducted mice experiments to verify the effect of LINC00511. Compared with others study the role of LINC00511 in tongue squamous cell carcinoma without vivo experiments [30], the data from our study stayed more convincing. The data collected from the tumor revealed that knockdown of LINC00511 could inhibit tumor growth and the volume and weight of tumor were evidently declined by sh-LINC00511#1. The expression of NFIX and Ki67 were also cut down by sh-LINC00511#1while the expression of miR-625-5p expression was enhanced.

## Conclusion

Taken together, all the results collected from our study manifested that LINC00511 was a tumor promoter to sponge miR-625-5p by targeting NFIX in GC cell. Knockdown of LINC00511 could be considered as a brand new target for GC treatment. We will explore the role of LINC00511 in clinic analysis in the future to fill the gap of our research weakness.

## Supplementary information

**Additional file 1: Fig.** **S1**. **a** LINC00511 expression was examined in I/II and III/IV stage of GC. **b** The expression of miR-625-5p was evaluated in vivo. *P < 0.05, **P < 0.01.

## Data Availability

Research data are not shared.

## Abbreviations

GC: gastric cancer
lncRNAs: long non-coding RNAs
ceRNA: competing endogenous RNA
miRNAs: microRNAs
EMT: epithelial mesenchymal transition
RISCs: RNA-induced silencing complexes
