# Supplementary figures and images for: LINC00511 accelerated the process of gastric cancer by targeting miR-625-5p/NFIX axis
Source: Cancer Cell Int. 2019 Dec 26;19:351. doi: 10.1186/s12935-019-1070-0 (PMC6933746; doi:10.1186/s12935-019-1070-0)

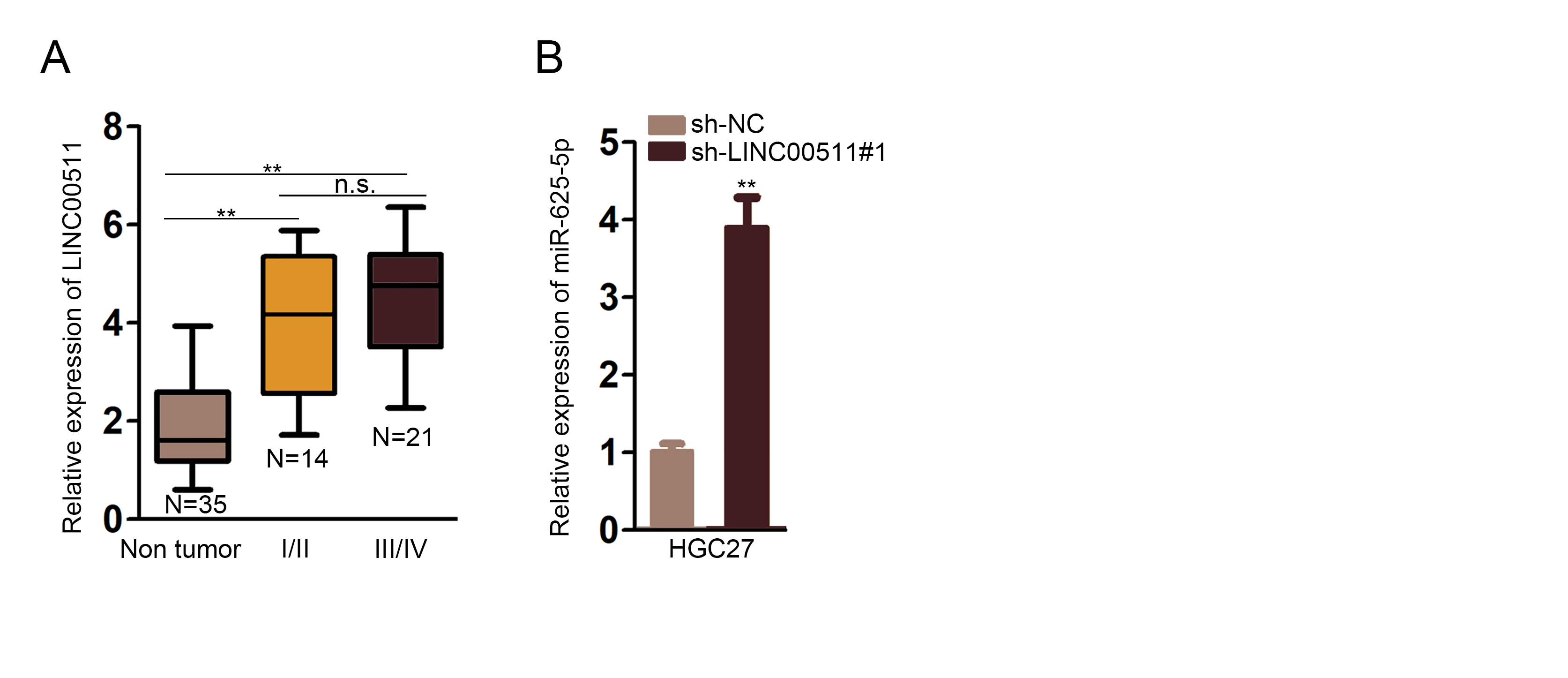

Supplement: Supplementary file 1 — Additional file 1: Fig. S1. a LINC00511 expression was examined in I/II and III/IV stage of GC. b The expression of miR-625-5p was evaluated in vivo. *P < 0.05, **P < 0.01. [file 12935_2019_1070_MOESM1_ESM.tif]
